# Facility-based surveillance for emerging infectious diseases; diagnostic practices in rural West African hospital settings: observations from Ghana

**DOI:** 10.1098/rstb.2016.0544

**Published:** 2017-06-05

**Authors:** Freya L. Jephcott, James L. N. Wood, Andrew A. Cunningham

**Affiliations:** 1 Department of Veterinary Medicine, University of Cambridge, Cambridge CB3 0ES, UK; 2 Institute of Zoology, Zoological Society of London, Regents Park, London NW1 4RY, UK

**Keywords:** International Health Regulations, Integrated Disease Surveillance and Response, febrile illness

## Abstract

The aim of this study was to better understand the effectiveness of Integrated Disease Surveillance and Response (IDSR) facility-based surveillance in detecting newly emerging infectious diseases (EIDs) in rural West African settings. A six-month ethnographic study was undertaken in 2012 in the Techiman Municipality of the Brong-Ahafo Region of Ghana, aimed at documenting the trajectories of febrile illness cases of unknown origin occurring within four rural communities. Particular attention was paid to where these trajectories involved the use of formal healthcare facilities and the diagnostic practices that occurred there. Seventy-six participants were enrolled in the study, and 24 complete episodes of illness were documented. While participants routinely used hospital treatment when confronted with enduring or severe illness, the diagnostic process within clinical settings meant that an unusual diagnosis, such as an EID, was unlikely to be considered. Facility-based surveillance is unlikely to be effective in detecting EIDs due to a combination of clinical care practices and the time constraints associated with individual episodes of illness, particularly in the resource-limited settings of rural West Africa, where febrile illness due to malaria is common and specific diagnostic assays are largely unavailable. The success of the ‘One Health' approach to EIDs in West Africa is predicated on characterization of accurately diagnosed disease burdens. To this end, we must address inefficiencies in the dominant approaches to EID surveillance and the weaknesses of health systems in the region generally.

This article is part of the themed issue ‘One Health for a changing world: zoonoses, ecosystems and human well-being'.

## 1. Introduction

Emerging infectious diseases (EIDs) represent a major threat to global health. In recent years, re-emerging and newly emerging wildlife-associated zoonoses such as Ebola virus in West Africa, Severe Acute Respiratory Syndrome (SARS), Human Immunodeficiency Virus (HIV) and numerous novel strains of influenza have led to substantial economic and human losses [1].

Prior to constituting a major outbreak, many zoonoses may exist within communities for some time as isolated or small clusters of cases [2,3]. Such cases represent an important opportunity for early intervention but often proceed undetected due to a range of poorly defined clinical and social factors, many of which are exacerbated by the remote and tropical environments in which wildlife-associated spillover events tend to occur. West Africa has been identified as an environment particularly prone to zoonotic spillover and as such requires special attention for this role in global health [4]. The recent West African Ebola epidemic exemplifies this.

The Integrated Disease Surveillance and Response (IDSR) programme is a Centers for Disease Control and Prevention (CDC) and World Health Organization (WHO) devised template for domestic infectious disease control infrastructure. In common with much of sub-Saharan Africa, West African countries rely on the IDSR to implement the revised International Health Regulations (IHR, 2005). The revised IHR include mandated surveillance and reporting requirements for ‘any event of potential international public health concern, including those of unknown causes or sources’ [5]. Previously, the IHR only required cases of three named diseases (cholera, plague and yellow fever) to be reported. Substantial changes to reporting and surveillance requirements were triggered by the international spread of SARS in 2002, a previously unidentified zoonosis.

The IDSR primarily relies on facility-based surveillance for the detection of individual or small numbers of cases [6]. This approach involves a healthcare worker, typically a doctor, identifying a significant disease within their normal professional activities treating patients. As often noted, rare or novel conditions such as an EID are difficult to detect through this approach, especially in a resource-limited clinical setting where there is a high burden of routine infectious diseases. Here, we elucidate some of the socio-medical mechanisms that have an impact on facility-based surveillance in an under-resourced rural West African setting. We explore clinical diagnostic processes and their implications for the unseen emergence of novel pathogens.

This context places this study of how novel zoonoses may be diagnosed (or not) at the centre of real world One Health issues; if diseases are not diagnosed, they will remain neglected. The assumption is often made that important zoonoses can be detected and responded to ‘at source’, although the lack of reporting of single cases or isolated clusters of important human diseases like Ebola is evidence that primary cases that do not spread are almost invariably missed. It is of great concern that current systems must be missing a significant burden of disease.

## 2. The study

For this study, we set out to explore the effectiveness of facility-based surveillance in rural West Africa by undertaking a 6-month ethnographic study aimed at documenting complete trajectories of cases of cryptic febrile illness arising in the rural community of Buoyem in the Techiman District of the Brong-Ahafo Region of Ghana.

Buoyem is a rural agricultural community comprising a central town with a population of approximately 4000 and a collection of around 20 smaller satellite villages accounting for another 5000 inhabitants. The study involved 76 participants recruited from nine households in the Buoyem area (table 1). Of the 76 participants enrolled, 31 came from three households in the main town and 45 from six households taken from peripheral villages selected for their progressive remoteness, as measured by distance to a paved road (an established determinant of formal healthcare utilization) [7]. Enrolled households were visited approximately twice a week for the duration of the fieldwork so that febrile illness episodes could be detected early on and followed in their entirety. During the course of the study, 24 of the 76 enrolled participants developed fevers of unknown origin and were thus incorporated into the research as case studies.

**Table 1.** Characteristics of participants at enrolment according to the village/town status of the household they belong to.

		town	villages	combined
number of households enrolled		3	6	9
household distance to paved road (minutes walking)		0–5	35–120	
number of participants		31	45	76
average household size		10	7.4	8.3
age of participants	adults (aged 18 years or over)	18	25	43
	children (below the age of 18 years)	13	20	33
health insurance status	number of participants insured at the time of the study	26	16	42
household tribal affiliation	Bono	3	4	7
	Fulani	0	1	1
	Mossi	0	1	1

The criterion for inclusion as a case study was a self-reported fever of unknown origin occurring within the previous 24 h. Fever (a body temperature exceeding 37.5°C) is a fairly universal symptom in response to infection. It was reasoned therefore that health-seeking behaviours in response to fever with any combination of other symptoms should provide a good insight into how EIDs might progress in a remote environment. When a participant self-reported a fever, they were observed throughout the illness episode with particular attention being paid to their health-seeking behaviours and, where the episode involved formal healthcare settings, the processes of nosology. In addition to participant observation in clinical settings, subsequent interviews with the involved healthcare workers and the collection of data from secondary sources, such as patient files and hospital records, took place.

Sixteen of the recorded illness episodes were deemed ‘routine’, meaning they were perceived as non-life-threatening and resolved within 10 days with only informal local treatment. This typically comprised the use of licensed pharmaceuticals leftover from previous illness episodes or purchased from a local drug seller, sometimes in combination with a homemade herbal preparation.

**Figure 1.**
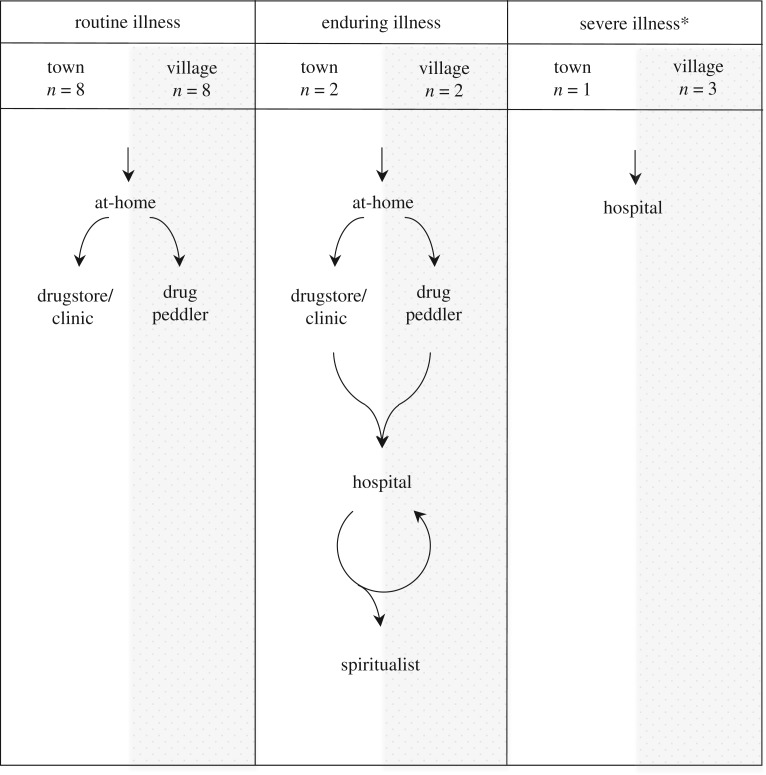
Participants’ patterns of treatment seeking according to the perceived severity of the illness episode and the village/town status of the household they belong to. Asterisk, in three of the four ‘severe illness’ case studies, an ‘enduring illness’ pattern of treatment seeking preceded the illness being classed as ‘life threatening’ and the ‘severe illness’ trajectory being initiated.

Of the eight participants who had illness episodes that were categorized as ‘severe’ or ‘enduring’, meaning they were perceived to be life-threatening or else failed to resolve within 10 days of informal treatment, all used a nearby hospital. Three participants belonging to town households attended the town's nurse-run clinic prior to presenting at a hospital (figure 1). Participants coming from village households tended to shun the clinic, claiming it was too costly relative to the effectiveness of the treatments available there. As all of the cases deemed ‘severe’ or ‘enduring’ ultimately ended up presenting to a hospital, shortcomings with facility-based surveillance are therefore likely not a consequence of the health-seeking behaviours of rural populations. Rather, if facility-based surveillance is not working, the dysfunction must be within the healthcare setting. As such, our discussion is focused on our observations of the nature and effect of the diagnostic processes within clinical settings.

Across the eight patients and illness episodes that involved hospital treatment and consultation with a doctor, the observed diagnostic processes were extremely diverse and apparently vulnerable to the interactions of numerous material and human factors. With little or no available literature on the drivers of diagnosis in resource-limited settings, a discussion, even one based on this limited sample, is insightful and important.

## 3. Diagnosis in context

As expected for any clinical setting, the doctors within the study reported employing a differential diagnostic approach to choosing treatment strategies for their patients. A differential diagnosis is commonly perceived as a systematic and exhaustive process. The three basic steps of a differential diagnosis are examining the patient, compiling a list of candidate conditions and testing candidate conditions in order of perceived likelihood until the underlying condition, or conditions, has been identified. What follows is a description of the most obvious impediments to accurate diagnosis of a patient presenting to a regional Ghanaian hospital with a febrile illness, organized in terms of the three primary steps of differential diagnosis. The following description reveals that, when performed in a resource-limited clinical setting, the diagnostic process is often neither linear nor conclusive.

## 4. Examining the patient

The first step in performing a differential diagnosis involves the physician gathering the relevant information about the patient and their condition. This often involves the taking of a medical history and sometimes a physical examination of the patient. In this study, however, this step was limited to a nurse measuring the patient's temperature, weight and blood pressure and the doctor prompting the patient for a list of their current symptoms. This limited interaction comprised all of the communication during the consultation for all of the eight case studies. All participants remained unaware of their diagnosis and the nature of the drugs that were prescribed, which is consistent with findings from other studies of Ghanaian clinical settings [8]. This lack of communication was associated with poor doctor–patient relationships and perhaps reflected a low doctor-to-patient ratio (about 22 doctors served a population of approximately 216 481 people in 2013) [9]. For seven of the eight cases, this lack of communication—in particular a tendency for patient records to be maintained but not reviewed—led to doctors unknowingly prescribing treatments that had already been prescribed to, and taken by, the patient following earlier visits to formal healthcare facilities, such as the hospital and town clinic. This resulted in therapeutically and diagnostically redundant visits and additional costs to the patient.

## 5. Compiling the list of candidate conditions

Following the assessment of the patient, the next stage of the differential diagnosis process involves the doctor compiling a list of possible candidate conditions. In theory, these are organized according to likelihood based on the presentation of the patient and the doctor's knowledge of local disease prevalence. In practice, all eight participants who presented to a clinic or hospital with a fever or a reported history of fever were initially diagnosed with, and treated for, malaria. No diagnostic tests, including malaria rapid diagnostic tests (RDTs), which were available at some sites, were employed. This practice of presumptive treatment of fever cases for malaria can be linked to a now superseded set of WHO 2010 guidelines on the treatment of malaria in children. However, a growing body of research suggests that it is still commonplace in clinical settings across much of Africa [10,11].

As revealed in interviews with the doctors, where antimalarial treatment failed to relieve the symptoms of the patient, the next recourse was the prescription of a broad-spectrum antibiotic. This unofficial treatment protocol was observed in five of the eight case studies. One doctor explained that this was due to the availability of broad-spectrum antibiotics and that it was often a successful strategy. Given that a primary role of doctors in this setting is to treat successfully as many patients as possible within severe material and time restraints, the identification of a particular disease aetiology is not always necessary or pursued.

## 6. The process of elimination

The presumptive diagnosis of malaria and subsequent presumptive diagnosis of bacterial infection were both hurdles for the consideration of other candidate conditions. These would not have caused such significant setbacks to the diagnostic process if the testing and discrediting of candidate diagnoses had been done with diagnostic tests and not through the trial and error of different treatments.

From the limited number of case studies presented, we cannot establish any patterns of utilization for the diagnostic tests available at the hospitals. There were significant financial disincentives to the use of diagnostic tests, however, both for the hospital and for the patient. Patients had to coordinate their own testing, often requiring multiple trips to the hospital at their own expense. Uninsured patients were required to pay for diagnostic tests, and the National Health Insurance Scheme (NHIS) would provide only limited reimbursement to the hospital for insured patients. The more-specialized tests required to identify any uncommon EID, should a relevant test exist, would be even less accessible and unlikely to be used.

It was apparent through the case studies that diagnostic technology is not routinely used when managing a febrile illness, at least not in the early stages. As this resulted in most candidate conditions being tested through the patient's response to various treatments, often only one diagnosis could be considered or discredited at each visit. This had particularly severe repercussions for the rural villagers seeking hospital treatment. The increased associated transport costs the villagers faced and the lower average income and likelihood of NHIS membership meant they had to source money communally for their trip and treatment. This typically limited them to no more than two hospital attendances per episode of illness.

Participants from the town experienced fewer financial restrictions than the villagers and were able to pursue hospital treatment across lengthy courses of illness. They typically only ceased hospital care when failure to resolve the illness led them to doubt the efficacy of the biomedical approach. It was often at this point that a participant would engage a professional traditional healer such as a spiritualist. Both the villagers and the townsfolk were largely unaware that many of their hospital visits were redundant. However, these repetitive and often unproductive visits were instrumental in the decision of both groups to not return to the hospital.

## 7. Final diagnoses

Within the differential diagnosis process, there was no mechanism for feedback to notify the doctor of a successful diagnosis and treatment of a patient. It was therefore impossible to differentiate between a successful and failed diagnosis. Correct diagnosis, spontaneous recovery, premature exit resulting from lack of funds or lack of faith in the biomedical approach or death all produced a final untested diagnosis. For example, one of the participants was diagnosed and treated for malaria twice at a hospital in the week prior to his admission as a suspected meningitis case. He died the day following admission. During his admission, a diagnosis of meningitis was rejected and replaced by a tentative diagnosis of hepatic encephalopathy that was not explored. As the illness episode concluded, this diagnosis was recorded as the cause of death without laboratory testing.

Another factor reducing any consideration of an uncommon diagnosis is the disproportionate representation of diseases perceived to be common locally. In the course of a single episode of illness, multiple disease labels were generated across multiple hospital attendances. For instance, in the case above, the hospital recorded two cases of malaria and one case of hepatic encephalopathy. Such a process distorts doctors' perceptions of local epidemiology by skewing disease surveillance data towards already common conditions and further prejudicing them against unusual or less well-established diagnoses.

The exact combination of factors that reduced the reliability of the differential diagnosis process varied between cases. In all eight cases, however, confounding factors were sufficient to precipitate an early departure from the process. Not one of the eight participants captured in the study ceased to pursue hospital treatment because they had been successfully treated.

## 8. The implications for early detection of emerging infectious diseases and possible alternatives

Many of the factors implicated in the consistent failure to diagnose participants are improving across Ghana. Healthcare and transport infrastructure are improving, there is a decreasing burden of many common infectious diseases and there are interventions aimed at improving doctor–patient relationships [12,13]. Many of the recurring problems in identifying early cases of EIDs are not unique to Ghana or West Africa. The diagnosis of a rare condition within the time restrictions of a single episode of illness is problematic in any clinical setting. This difficulty is due primarily to the differential diagnosis process being based on a likelihood model and the uncommon (by definition) nature of EIDs. The processes of identifying and labelling diseases via clinical diagnosis therefore make facility-based surveillance unsuitable as a primary source of EID surveillance.

The use of facility-based surveillance for EIDs within the IDSR framework is likely by default rather than by design. The IDSR technical guidelines were originally developed to tackle major burdens of infectious disease and, as such, do not include specific instructions for EID surveillance. This results in the task of EID surveillance being absorbed by the system in place for identifying and controlling common infectious diseases. As the IDSR is the elected vehicle for the revised IHR (2005) in most of Africa, an effective EID surveillance system needs to be developed, especially in environments prone to EIDs.

A more suitable alternative to facility-based surveillance could be the establishment of specialized national diagnostic laboratories that are able to receive and test samples without the patient or local health clinic incurring additional costs or crippling bureaucracy. A number of studies have shown that the existence of a previously unknown pathogen within a human population often does not signal an impending pandemic. Indeed, some novel zoonoses have been found to cause only a single case or a small number of cases before apparently disappearing from the population entirely [14–16]. Taking a slower approach to detection, one that exceeds the length of an episode of illness, might be a possible solution. A similar approach is already employed in Ghana for influenza surveillance, where regional hospitals act as sentinel sites routinely sending samples to a specialized laboratory to monitor the strains circulating within the country.

An archival approach to EID surveillance has utility by slowly contributing to a more nuanced knowledge of the local epidemiology. This characteristic, however, is at the expense of the immediate utility promised by facility-based surveillance in containing the threat of an outbreak, a function it may serve in response to larger and more sudden spillover and outbreak events. As such, the introduction of a laboratory-based system for EID detection should not usurp the place of the facility-based system within the IDSR framework but, rather, complement it by providing a more systematic and reliable approach to surveillance. Such a reformed system needs to be targeted at regions, such as Central and West Africa, which are particularly susceptible to zoonotic spillover and therefore likely to see the emergence of a new infectious disease.

Regardless of the exact approach taken, the creation and incorporation of a dedicated system of EID surveillance into African countries’ national disease control infrastructure is imperative. This is not to say that there is not also a need to address the various factors confounding healthcare providers’ use of differentials diagnoses in resource-limited settings, which are crucial to the delivery of effective clinical care. However, the success of the One Health approach to EIDs in West Africa is predicated on characterization of accurately diagnosed disease burdens. We must attend, therefore, to the inefficiencies in our dominant approaches to EID surveillance in West Africa or we will be unable to effectively set public health priorities and prevent future disease outbreaks such as the recent Ebola epidemic.
